# Integrated sequence and expression analysis of ovarian cancer structural variants underscores the importance of gene fusion regulation

**DOI:** 10.1186/s12920-015-0118-9

**Published:** 2015-07-17

**Authors:** Vinay K. Mittal, John F. McDonald

**Affiliations:** Integrated Cancer Research Center, School of Biology, and Parker H. Petit Institute of Bioengineering and Biosciences, Georgia Institute of Technology, 315 Ferst Dr., Atlanta, GA 30332 USA

## Abstract

**Background:**

Genomic rearrangements or structural variants (SVs) are one of the most common classes of mutations in cancer.

**Methods:**

An integrated DNA sequencing and transcriptional profiling (RNA sequence and microarray gene expression data) analysis was performed on six ovarian cancer patient samples. Matched sets of control (whole blood) samples from these same patients were used to distinguish cancer SVs of germline origin from those arising somatically in the cancer cell lineage.

**Results:**

We detected 10,034 ovarian cancer SVs (5518 germline derived; 4516 somatically derived) at base-pair level resolution. Only 11 % of these variants were shown to have the potential to form gene fusions and, of these, less than 20 % were detected at the transcriptional level.

**Conclusions:**

Collectively our results are consistent with the view that gene fusions and other SVs can be significant factors in the onset and progression of ovarian cancer. The results further indicate that it may not only be the occurrence of these variants in cancer but their regulation that contributes to their biological and clinical significance.

**Electronic supplementary material:**

The online version of this article (doi:10.1186/s12920-015-0118-9) contains supplementary material, which is available to authorized users.

## Background

Cancer genomes are characterized by the presence of several classes of somatic mutations including point mutations, copy number alterations and chromosomal rearrangements or structural variants [1, 2]. Of these, SVs are the most frequent [3–5] and include tandem-duplications, inversions, deletions, insertions and inter-chromosomal translocations [1]. Although cancer genomes may harbor hundreds to thousands of SVs, only a handful are considered of potential functional significance, typically involving protein-coding genes [6–8]. Functionally significant SVs often involve gene fusions that place protein-coding genes under novel regulatory controls and/or result in the generation of novel fusion proteins [9–11]. A well-known example is the reciprocal translocation between chromosome 9 and 22 resulting in expression of the BCR-ABL fusion protein in chronic myeloid leukemia [12–14].

Advances in the application of the paired-end (or mate-pair) approach to high-throughput sequencing have made genome-wide surveys of genomic rearrangements possible [6, 15–18] and recent studies have uncovered a number of new gene fusions and other SVs of potential functional significance in a variety of cancer genomes [7, 8, 19–21]. However, the potential importance of gene fusions and other SVs to cancer onset and progression may be modulated if these variants are not expressed. Indeed, recent studies have revealed that “normal” tissues can harbor transcriptionally repressed “pro-neoplastic” SVs that only become oncogenic when transcriptionally activated [22]. Thus, to fully evaluate the functional significance of gene fusions and other SVs in cancers, DNA sequence analyses should ideally be coupled with transcriptional profiling. In an effort to address this issue, we utilized an integrated computational workflow to analyze DNA sequencing and transcriptional profiling (RNA sequence and microarray gene expression data) data from six ovarian cancer patient samples. In addition, DNA sequence data from matched sets of control (whole blood) samples from these patients were used to distinguish cancer SVs of germline origin from those arising somatically in the cancer cell lineage. We report here the detection of 10,034 ovarian cancer SVs (5518 germline derived; 4516 somatically derived) at base-pair level resolution. Only 11 % of these variants were found to have the potential to form gene fusions and, of these, less than 20 % were detected at the transcriptional level. Collectively, our results demonstrate the presence of large numbers of germline and somatically derived gene fusions and other SVs in ovarian cancer tissues and underscores the potential importance of the regulation of gene fusions in cancer onset and progression.

## Methods

### Sequencing data acquisition

All specimens were obtained from patients with appropriate consent from the relevant institutional review board. Whole genome sequencing (WGS) data for six ovarian serous cystadenocarcinoma and matched somatic control (whole blood) samples were selected from The Cancer Genome Atlas (TCGA) data portal [23] using dbGAP in BAM file format. These six patients were selected because DNA-Seq, RNA-seq and gene expression microarray data were available for each of these patients. The WGS data consisted of 22 billion (range 1.3–2.54 billion) 75–100 bp long paired-end reads (in forward-reverse orientation) generated from the Illumina GAII instrument. The RNA-Seq data consisted of 1 billion (range 105–243 million) 75 bp long paired-end reads also generated from the Illumina GA II system. Samples and sequencing data are summarized in Additional file 1: Table S1. BAM files containing the sequencing data were sorted using Picard Tools [24] SortSam and converted to FastQ format using BamToFastQ program [25].

### Genomic SV detection using WGS

Massive amounts of whole genome sequencing data present a challenge in detecting complex structural variants with high-accuracy. In order to accurately detect and characterize genomic structural variants, we designed a streamlined workflow (summarized in Additional file 2: Figure S1) as follows:

#### SV detection

The overall quality of the WGS reads was assessed using FastQ. Low quality (Q < 20) bases and adapter sequences were removed from the ends of the reads. The remaining reads were aligned to the human reference genome hg19 (GRCh37 assembly, UCSC genome database) using Bowtie2 [26]. Unmapped reads were stored in a separate file. PCR duplicates were removed from the alignment files using the Picard Tools [24] MarkDuplicate program. Since highly repetitive/low complexity genomic regions may result in ambiguous or low confidence alignments, we conservatively filtered out all alignments with mapping quality (MAPQ) < 35. Various classes of large structural variants (SVs) were detected using SVDetect [27]. The SVDetect algorithm searches for clusters of paired-end reads creating distinct signatures of structural variants in the alignment file. SV signatures are called if any or both of the inherent characteristics of paired-end sequencing constraints (*i.e.*, library insert-size, alignment orientation of mates relative to each other) are violated. Based on the clusters of paired-end signatures, the SV calls are generated and the location of breakpoints is estimated. Genomic loci involved in a SV (a.k.a.“links”) are required to be supported by a minimum number of paired-end reads as determined by the sequencing depth of coverage [28]. Since our samples have different sequencing depths of coverage, different cutoff values were determined for each sample (Additional file 3: Table S2).

#### Filtering

We observed that more than 50 % of the SV calls were ‘small_duplications’ that could be the result of artifacts generated during the library preparation. Thus, we conservatively removed such calls as well as those described as ‘co-amplicons’ and ‘undefined’ calls generated from ambiguous paired-end signatures. We additionally removed all SV calls that had more than 50 % overlap with the low-complexity genomic regions. Since, reference human genome quality is questionable around the centromere and telomere regions and near the assembly gaps, we also removed SVs mapping within 100-kilo base-pairs of these regions. Finally, we removed all called SVs that mapped either to mitochondrial or Y chromosome or currently un-localized regions of the genome (“Un”, “hap”, *etc.*).

#### Targeted assembly of SVs

The paired-end read approach for SV detection does not provide base-pair level breakpoint information but rather provides genomic regions that may contain potential breakpoints. Also, short (75 – 100 bp) read mapping to the reference genome may generate false clusters of paired-end reads resulting in false SV calls. In order to confirm SV calls generated by SVDetect and to establish breakpoints at the base-pair resolution, we performed a targeted *de novo* assembly for each SV call. *De novo* assembly is performed by progressively merging redundant DNA sequences with shared overlapping ends determined by a pre-specified parameter called ‘k-mer’ length. The goal is to reconstruct the exact DNA sequence underlying the SV. For the assembly of each SV call, we included sequencing reads mapping within the 500 bps on either side of the genomic regions involved in a SV call. Also included are reads that initially could not be mapped to the reference genome. Since a complete assembly cannot be achieved using single k-mer length, we performed multiple k-mer length assemblies by varying k-mer from half of the read length (37–50 bp) to the complete read length (75–100 bps) in 2 bp increments. Multiple k-mer assemblies were performed using ABySS [29] and later merged using Trans-ABySS [30] that also removes redundant assembled sequences from the assembly. In order to further expand the assembly set, we performed multiple k-mer assemblies using an additional assembly program, Velvet [31].

#### Validation and breakpoint detection

Assembled DNA sequences (also called contigs) were mapped to the human reference genome (hg19) using the BWA [32] program that independently aligns sections or fragments of a DNA sequence to discrete genomic loci. Such mapping is called split-mapping. Genomic coordinates of the paired-end based SV calls from SVDetect were compared with the fragmented alignments of the assembled contigs and breakpoints were determined for SV calls supported by the assembly. For each SV, two breakpoints are detected each corresponding to the genomic locus participating in the SV formation. Validation and breakpoint detection were carried out using our previously developed pipeline, R-SAP (RNA-Seq analysis pipeline; [33]), that accurately detects fragmented alignments (split-mapping) representing potential gene fusions and/or genomic rearrangements. R-SAP modules were slightly modified to include intragenic SVs such as deletions and insertions and other complex SV signatures such as transpositions that were not detected in the original R-SAP configuration. In order to minimize false SV calls supported by the assembly, the assembled contigs were aligned to the reference human genome using an additional alignment algorithm SSAHA2 [34], and the SVs again validated using R-SAP. The result is a stringently defined set of SVs with breakpoints detected at the base-pair level resolution.

### Fusion transcript detection using RNA-Seq

RNA-Seq reads obtained from TCGA were initially subjected to quality and adapter filtering using FastQC and TrimGalore [35]. Reads were subsequently aligned to the reference human genome in paired-end mode using TopHat (‘fusion-search’ mode). TopHat-fusion [36] searches for potential breakpoints using the ‘split-read’ alignment of sequencing reads were supported by additional ‘paired-end’ reads. Potential fusions reported by TopHat were further validated using reference transcript annotations established as a merged set of Ensembl (version 73) and lncRNAs available from the UCSC genome database [37–39]. Finally, we required that each fusion be supported by at least one split-read and one paired-end read alignment. We conservatively discarded fusions where both ends of the fusion were confined to single gene loci.

### Gene-expression analysis

We downloaded gene-expression microarray data for the same 6 ovarian cancer samples using the TCGA data portal. Due to the unavailability of gene expression data from matched normal samples from the cancer patients examined, we utilized as controls, the results of assays carried out on normal ovarian tissues collected from eight healthy (age matched) women. These data were also downloaded from the TCGA portal. The microarray expression data were generated by RNA hybridization to Affymetrix HT_HG-U133A gene chips and data files were provided in ‘cel’ format. Expression values were estimated and normalized by the RMA normalization method from the cel files using the Affymetrix Expression Consol [40]. For each gene, the average expression values across all of the eight normal samples were used as the denominator (numerator was expression values determined for each cancer sample) in computing the gene expression fold-change across the ovarian cancer samples.

## Results

### DNA sequence analyses

#### More than 10,000 structural variants (SVs) were identified in the six ovarian cancer patient samples

DNA sequencing data of matched sets of six ovarian serous cystadenocarcinoma and six somatic control (whole blood) tissues (Additional file 1: Table S1) were downloaded from the The Cancer Genome Atlas data portal [23] using dbGAP in BAM file format. The raw data consisted of 22 billion 75–100 bp paired-end reads (range 1.3–2.54 billion; Additional file 1: Table S1). An integrated computational workflow was developed to facilitate the data analysis (Additional file 2: Figure S1; see Methods for details).

**Fig. 1:**
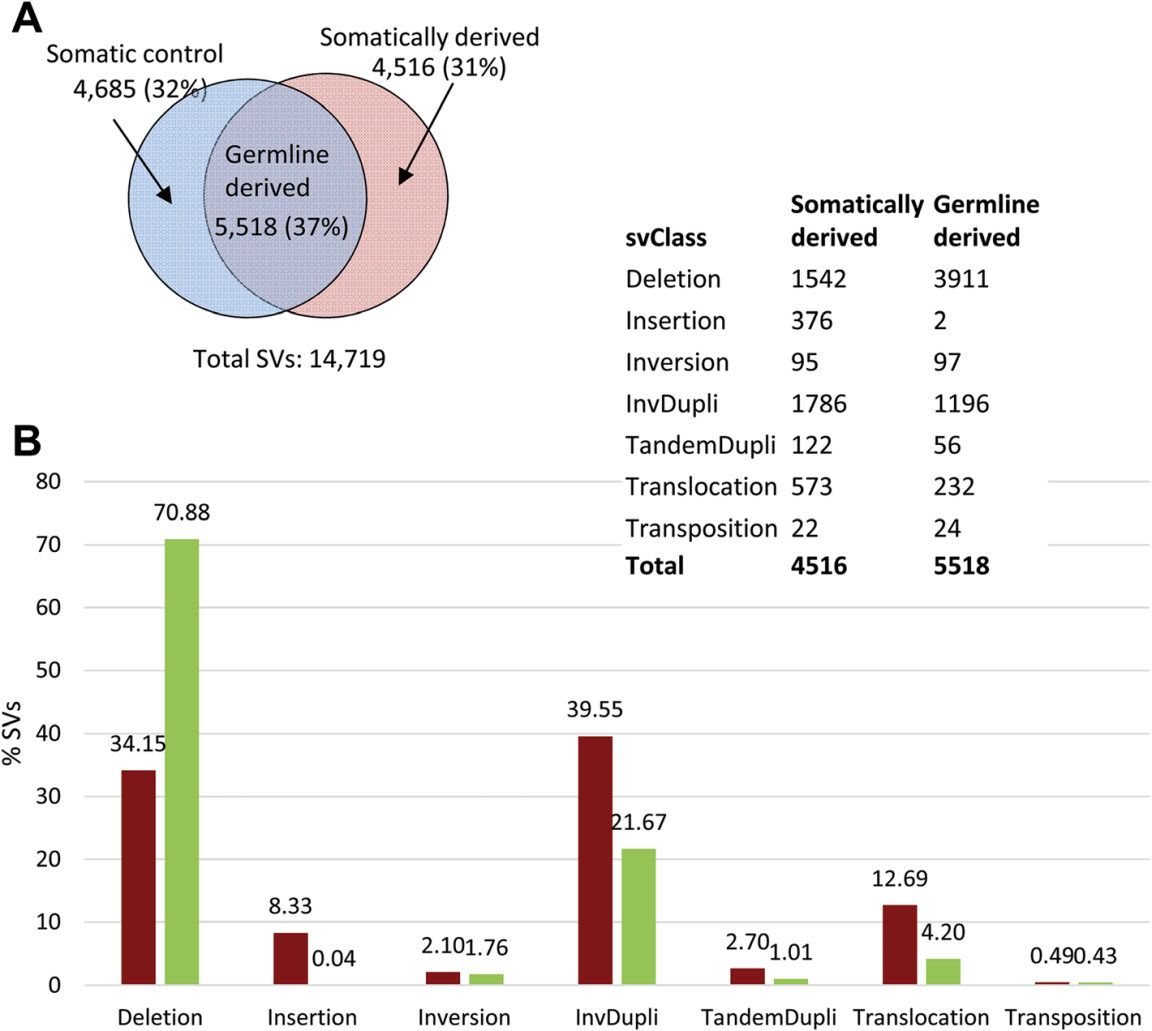
**a** The number (percentage) of germline and somatically derived ovarian cancer SVs in six ovarian cancer patient samples. Circles represent the total number of SVs detected in somatic control (whole blood; blue circle) and cancer (red circle) tissue samples collected from six ovarian cancer patients. SVs identified in both the control and cancer samples were classified as germline derived, while SVs detected in only the cancer samples were classified as somatically derived. **b** Distribution of SVs across structural categories. Somatically derived (red) and germline derived (green) SVs were further categorized according to the underlying genomic rearrangement. Deletions were the most abundant category accounting for the majority (~71 %; see percentages on top of bars) of the germline derived SVs. Corresponding data are shown in the Table (top right corner). InvDupli: inverted-duplications, TandemDupli: tandem-duplication

Initial alignments resulted in the mapping of 84 % of the DNA-Seq reads to the human reference genome (hg 19). A subsequent series of stringent filtering and validation steps (see Methods) resulted in a total of 35,721 SV calls. To confirm these SVs and to determine breakpoints at base-pair level resolution, targeted *de novo* assembly was performed for each SV call (Additional file 2: Figure S2). After correcting for multiplicity of confirmed SVs (presence of the same SV across multiple samples), a total of 14,719 unique SVs were detected across all samples (Additional file 4: Table S3). Of these, 32 % (4,685) were uniquely present in the somatic control (blood) samples, 31 % (4,516) were uniquely present in the cancer samples and 37 % (5518) were present in both the control (blood) and the cancer samples (Fig. 1a; Additional file 2: Figure S3). We classified SVs detected in both the cancer and somatic control (blood) samples as germline derived cancer variants since the presence of precisely the same SV in divergent somatic cell types implies a common clonal (germline) origin. Those SVs detected exclusively in the cancer samples were classified as somatically derived cancer variants arising in the cancer cell lineage. By these criteria, 5518 or 55 % of the 10,034 SVs detected in the cancer samples were germline derived and 4516 or 45 % were somatically derived.

The SVs detected in the cancer samples were comprised of seven structural classes: inversions, transpositions, tandem-duplications (100–10 million bps in size), deletions (>20 bps), insertions (>20 bps), inverted-duplications, and translocations. The distribution of these SVs across all cancer samples is summarized in Fig. 1b. The most frequent class of SVs was deletions and germline derived deletions were twice as frequent than somatically derived deletions. Germline and somatically derived inversions and transpositions were present in approximately equal frequencies while somatically derived SVs were more frequent than germline derived variants for all other classes of SVs (insertions, inverted-duplications, tandem duplications and translocations).

Mutations in BRCA1 and/or BRCA2 genes are known to be associated with genomic instability due to deficiencies in homologous recombination repair [41]. While we detected no BRCA mutations within the germlines of the patients analyzed in this study, the tumor tissues of two patients displayed somatic mutations in BRCA2 (Patient P2: frame shift DEL; Patient P5: missense mutation). However, we detected no significant elevation in the overall number of SVs between patients P2 and P5 and the other patients analyzed in the study.

**Fig. 2:**
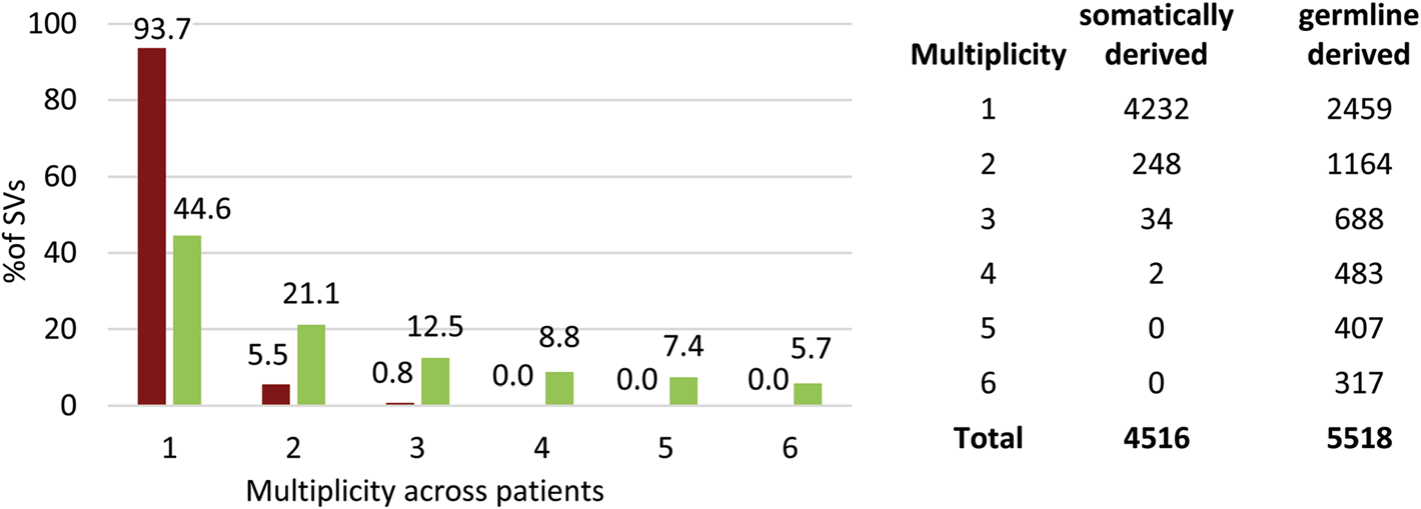
Multiplicity of SVs across samples. The X-axis represents multiplicity (number of occurrences) of somatically derived (red bars) and germline derived (green bars) SVs across cancer samples. The Y-axis represents percentage of SVs present in a particular multiplicity. The table presents the numerical distribution of SVs across the cancer samples

Analysis of the frequency of recurrence of SVs across the cancer samples indicates that, as expected, germline derived SVs occur across multiple samples at the highest frequency (Fig. 2). Indeed, more than half (3059/5518 or 55 %) of the germline derived SVs are present in multiple cancer patient samples reflecting naturally occurring variation in the human population. In contrast, only 6 % (284/4516) of the somatically derived SVs were detected in more than one cancer patient sample. These SVs could represent identical somatic mutations that have arisen recurrently across multiple OC patients or, perhaps more likely, variants that are segregating in the natural population but were in such low frequency in our germline samples as to go undetected in the sequencing analysis.

#### Ovarian Cancer SVs can be divided into 3 groups based upon the location of chromosomal breakpoints

**Fig. 3:**
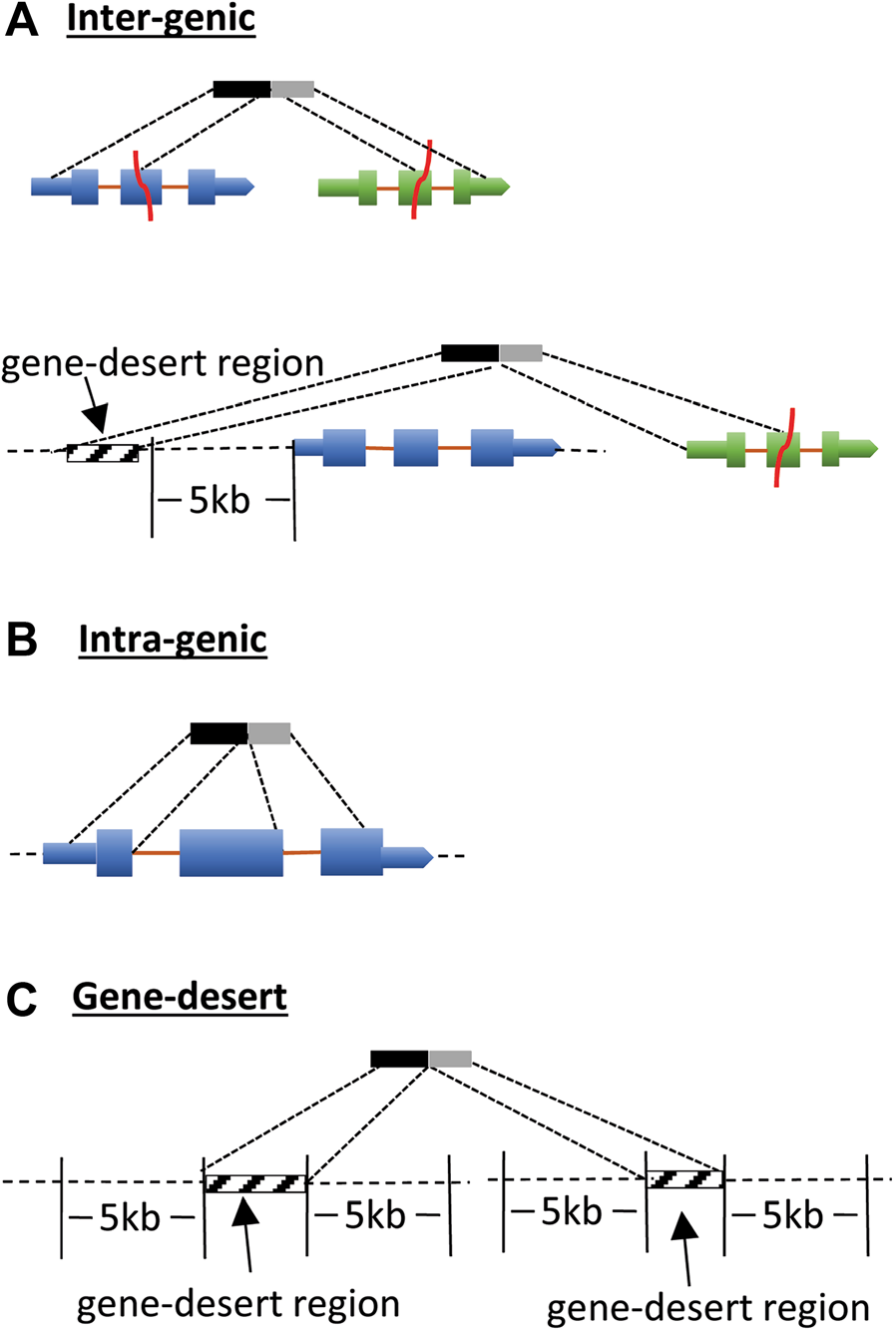
Classification of ovarian cancer SVs based on breakpoints. SVs are depicted by black-grey boxes, reference transcripts are represented by the blue and green boxes. Thick boxes represent open-reading-frame or coding sequences (CDS) while thin boxes represent UTRs. **a** Intergenic SVs – breakpoints map to annotated genes located at distant genomic locations; (**b**) intragenic SVs- breakpoints map within the same gene; (**c**) gene desert- breakpoints map to un-annotated genomic regions (gene deserts)

**Fig. 4:**
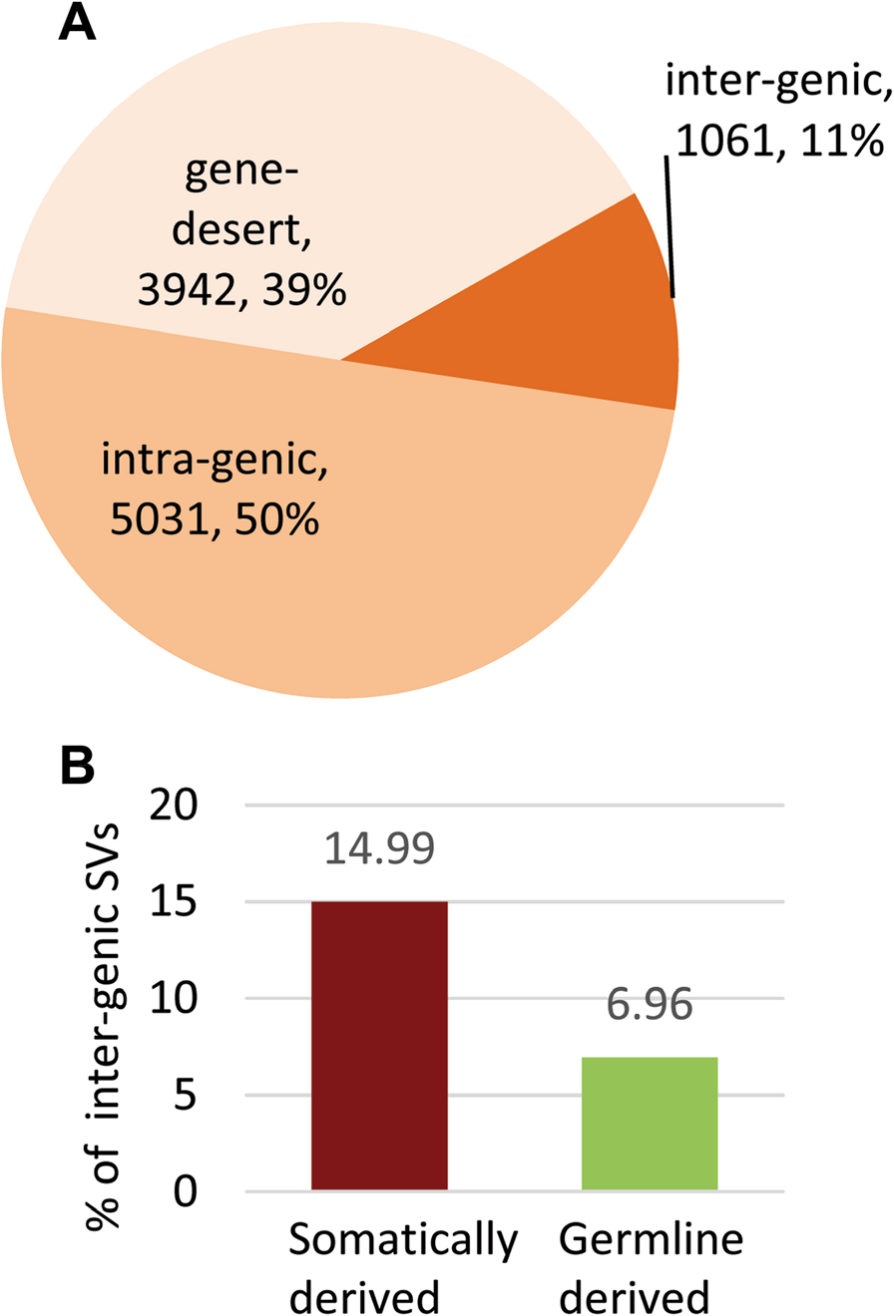
Distribution of SVs across classes. **a** Distribution of total detected SVs among classes. **b** Distribution of intergenic SVs between somatically derived and germline derived SVs. Intergenic SVs are significantly enriched (*p*-value < 0.05) for somatically derived variants

Detected SVs were annotated using a combined set of 224,555 normal reference transcripts (Ensembl annotations, release 73 and lncRNAs from the UCSC genome database). We classified SVs detected in our cancer samples into three groups based on the location of breakpoints relative to the reference transcripts as follows (Figs. 3 and 4a; see also Additional file 2: Figure S4): intergenic SVs are defined as variants with breakpoints mapping to two genes at distant genomic locations and at least one that overlaps with an annotated gene; intragenic SVs are variants with breakpoints mapping within a single annotated gene; and gene-desert SVs are variants with breakpoints mapping to distant locations within genomic regions devoid of annotated genes (“gene deserts”). Intragenic SVs are the most abundant class (50 %; 5,031/10,034) followed by gene-desert (39 %; 3,942/10,034) and intergenic (11 %; 1,061/10,034) SVs. Intergenic SVs are >2X more abundant among somatically derived variants than among germline derived variants (677 vs. 384; Fig. 4b) while the number of intragenic (2,151 vs. 2,880) and gene-desert SVs (1,688 vs. 2,254) display more similar in frequencies among somatically derived and germline derived variants.

#### Intergenic SVs encompass multiple classes of fusion-genes

We further divided intergenic SVs based on the location of breakpoints within the various gene regions, *i.e.,* the promoter region (defined as breakpoint regions within 5 kb up-steam of the transcriptional start site), the 5′ and 3′ untranslated leader regions (UTRs), and the protein coding sequence (CDS). Intergenic SVs where the 5′- partner gene sequence is fused with either a non-protein coding gene (*e.g.*, lncRNA) or with an unannotated region of the genome (gene-desert) are classified as 5′ truncated SVs. Finally, intergenic variants that do not manifest canonical gene structures (5′UTR-promoter-CDS-3′UTR), display gene components in incorrect orientation (*e.g.*, 5′ UTR-CDS-promoter-3′UTR; gene desert-3′ CDS, *etc.*) or otherwise cannot be functionally evaluated were classified as uncharacterized RNA (Additional file 5: Table S4).

**Fig. 5:**
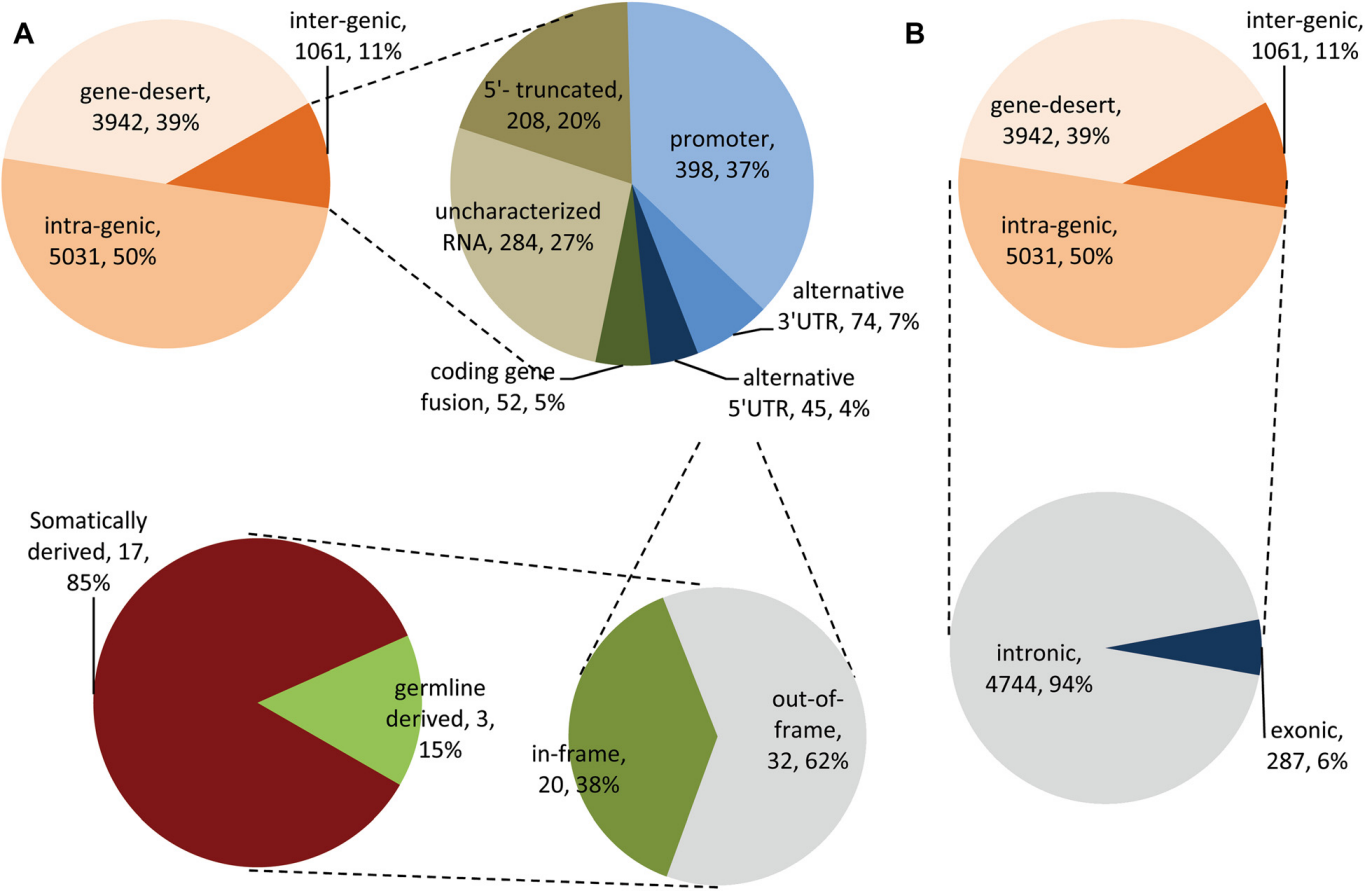
Distribution of intergenic and intragenic ovarian cancer SVs. **a** Distribution of germline and somatically derived intergenic SVs; (**b**) Distribution of intronic and exonic intragenic SVs

The relative distribution of these sub-classes of intergenic SVs in the cancer samples is shown in (Fig. 5a). The most abundant (37 %, 398/1061) sub-class of intergenic variants is associated with alterations in the promoter region of genes. These altered promoter variants along with the less frequent alternative 5′ UTR (4 %, 45/1061) and 3′ UTR (7 %, 74/1061) sub-classes all have the potential to alter the expression of associated genes without altering coding sequences. Intergenic SVs associated with the coding regions of genes also have the potential to alter the expression levels (*e.g.*, the 5′ partner gene typically provides the promoter region in addition to 5′ coding sequences while the 3′ partner may bring novel microRNA binding sites in its 3′ UTR) but may also generate novel fusion proteins if reading frames are maintained. While only 5 % (52/1061) of the intergenic SVs involve the fusion of coding regions of different genes, 38 % (20/52) of these variants were in frame. Interestingly, the vast majority of the in-frame gene fusions (85 %, 17/20) were somatically rather than germline derived suggesting that these *de novo* SVs have either been selectively favored in the cancer cell lineages or selected against in germline lineages or both. The majority of the coding region inter-gene fusions (62 %, 32/52) were out-of-frame.

#### The breakpoints of most intragenic SVs map to introns

The intragenic class of SVs was sub-divided into those with breakpoints mapping completely within the same intron (intronic) and those where at least one breakpoint mapped to an exon (exonic). The vast majority of intra-genomic SVs (94 %, 4,744/5,031) were intronic (Fig. 5b; Additional file 6: Table S5). Although intronic variants may affect splicing functions, they do not affect coding regions *per se*. Only 6 % (287/5,031) of the intragenic SVs grouped into the exonic sub-class. The majority of these exonic variants (138/287 or 48 %) mapped to non-coding genes (*e.g.*, lncRNAs) and are thus of currently undefined significance. The breakpoints of the remaining exonic variants mapped predominantly within 5′ or 3′ UTRs (5′UTRs: 17/287 or 6 %; 3′UTRs: 64/287 or 22 %). These intragenic variants could potentially alter regulatory sequences involved in gene expression (*e.g.*, upstream regulatory sequences in 5′UTRs or microRNA binding sites in 3′UTRs). The breakpoint of 24 % (68/287) of the exonic variants mapped to coding regions (CDS), which are presumed to disrupt the ORF and are labeled “disruptive” (Additional file 6: Table S5).

#### Many of the SVs detected in ovarian cancer map to gene desert regions

Although nearly 39 % (3,942/10,034) of the detected SVs were classified as gene-desert variants, their potential functional significance cannot be reliably inferred since the structure of transcriptional units within gene-desert regions is currently unknown.

### Gene expression analyses

#### A minority of intergenic SVs is transcribed

Intergenic SVs have the potential to generate functionally significant gene fusions. Thus, in an effort to explore the extent to which this class of SVs was being expressed in our cancer samples, we downloaded from the TCGA data portal the results of RNA sequencing (RNA-Seq) and microarray (Affymetrix) profiling studies carried out on these same samples. The raw RNA-Seq data consisted of 1 billion 75 bp paired-end reads (range 105–243 million) (Additional file 1: Table S1). These data were again analyzed using the computational workflow outlined in Additional file 2: Figure S1 (see Methods for details).

Since intergenic SVs with breakpoints mapping to the promoter region (398/1061) cannot be qualitatively distinguished using the RNA-Seq data, they were not part of the RNA-Seq analysis but are included in the microarray expression analysis described below. All other classes of intergenic SVs (coding region, 5′ truncated, alternative 5′ UTR, alternative 3′ UTR and uncharacterized RNAs) are included in the RNA-Seq analysis. The breakpoints of these fusion transcripts were detected in the RNA-Seq data using split-read mapping (see Methods). Since introns are spliced out during mRNA processing and are thus absent in the RNA-Seq data, we adjusted intronic SV breakpoints to the closest exon included in the gene fusion. SVs were categorized as detected by RNA-Seq if transcripts were found in at least 1 of the 6 cancer samples examined. Based on this criterion, 16 % (103/663) of these potential gene fusions were found to be expressed in the cancer samples. The percentage of the transcribed germline derived fusions (19 %, 30/158) was slightly higher than the somatically derived fusions (15 %, 73/505) (Table 1; Additional file 1: Table S1 and Additional file 7: Table S6).

**Table 1:** Summary of the number of the various types of SVs detected in the DNA sequencing analysis and their expression as detected by RNA-Seq or microarray studies (see text for details)

	SVs (DNA level)	Detected by RNA-Seq	Differentially expressed (microarray)
**A:** Somatically derived			
5′-truncated	167	29	NA
coding-gene-fusion	45	8	NA
Uncharacterized RNA	216	33	NA
alternative 5′UTR	30	1	7
alternative 3′UTR	47	2	14
Promoters	172	NA	21
**Total**	**677**	**73**	**42**
**B:** Germline derived			
5′-truncated	41	10	NA
coding-gene-fusion	7	0	NA
Uncharacterized RNA	68	14	NA
alternative 5′UTR	15	1	5^a^
alternative 3′UTR	27	5	4^a^
Promoters	226	NA	16
**Total**	**384**	**30**	**25**

^a^Overlap between RNA-Seq and microarray detection

#### Only somatically derived coding sequence gene fusions are expressed

**Fig. 6:**
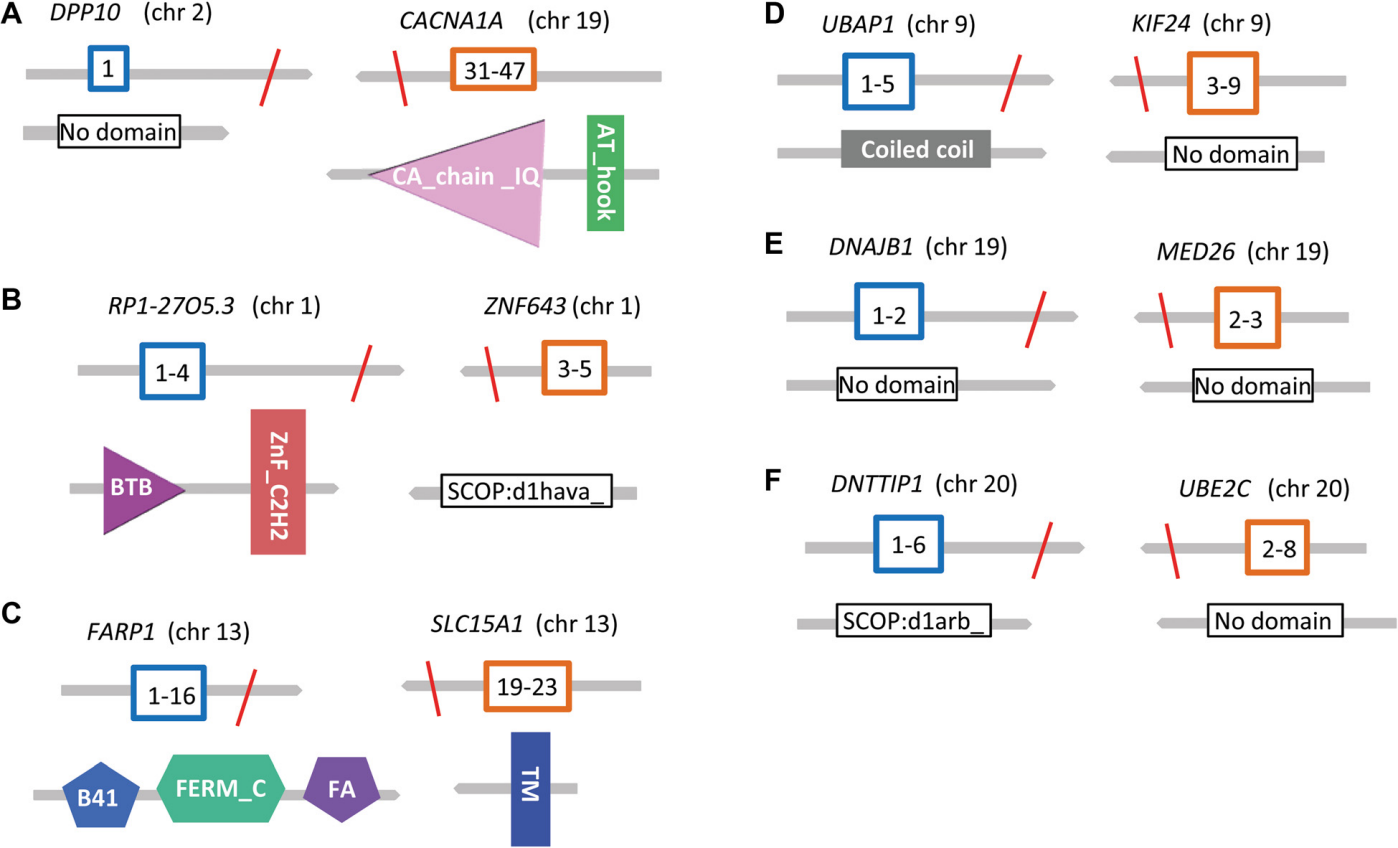
Structure of six intergenic SVs resulting in “in-frame” coding gene fusions. The figure represents the structure of the gene fusion and associated protein domains. Square boxes with numbers represent exons (5′ partner gene: blue, 3′ partner gene: orange), red lines represent the fusion breakpoint, gene symbols and corresponding chromosomes (in parenthesis) are shown on top of each gene fusion structure)

Of the 45 coding-gene fusions identified by the DNA-Seq analysis, only 8 were detected by RNA-Seq and all of these fusions were somatically derived (Table 1). Six of the 8 expressed fusions were in frame. In-frame fusions typically result in novel-fusion proteins that bring different protein domains together. We analyzed the rearrangement of protein domains resulting from the 6 in-frame gene fusions using the SMART database [42]. The fusion-gene structures and protein domain arrangements are shown in Fig. 6.

Two of the six in-frame gene fusions, *FARP1-SLC15A1* (13q32.2 inversion) and *RP1-27O5.3*–*ZNF643* (1p34.2 - 1p35.1 deletion), resulted in a novel juxtaposition of protein-coding domains. The domains associated with the *FARP1-SLC15A1* fusion are involved in a variety of signal transduction pathways that have previously been shown to influence cell-cell adhesion, cell migration and morphogenesis [43]. Similarly, the BTB/POZ domain (Broad-Complex, Tramtrack and Bric-a-brac) contained within the region of the *RP1-27O5.3* gene involved in the *RP1-27O5.3–ZNF643* fusion has been previously implicated in ovarian cancer growth and recurrence [44]. The potential functional significance of the remaining 4 somatically derived coding region gene fusions is currently unknown (Fig. 6). Other classes of transcribed intergenic SVs include alternative 5′ UTRs (4 %, 2/45), alternative 3′ UTRs (9 %, 7/74), 5′-truncated (19 %, 39/208), and uncharacterized RNAs (17 %, 47/284) (Table 1; Additional file 7: Table S6).

### Microarray analysis

Intergenic SVs with breakpoints mapping within the promoter region or within the 5′ or 3′ UTRs leave coding regions unchanged but may be expected to alter patterns of gene expression. In order to search for possible quantitative changes in expression associated with these intergenic SVs, we utilized the results of gene-expression microarray (Affymetrix HT_HG-U133A) analyses downloaded from the TCGA data portal for the same 6 ovarian cancer patient samples (Note that the 5′-truncated, coding-region and uncharacterized RNA fusions are not distinguishable in microarray studies and thus were analyzed exclusively in the RNA-Seq analyses discussed above). Since gene expression profiles of normal ovarian tissue from these patient samples are not available, we downloaded and utilized as controls the results of gene expression microarray profiles of normal ovarian tissue from 8 other age-matched women (Additional file 8: Table S7). Expression values were computed and normalized using the RMA normalization of the cel file data employing the Affymetrix Expression Consol. Fold-change in expression relative to the average of the 8 normal samples was computed for each of the genes associated with intergenic altered promoters, as well as altered 5′ and 3′ UTRs SVs (Additional file 7: Table S6). Fold changes > 2X between control and cancer samples were considered significant. The numbers of SVs displaying significant changes by this criterion are displayed in Table 1.

Of the 517 (249 somatically derived and 268 germline derived) gene fusions (promoter, alternative 5′UTR and alternative 3′ UTR only) analyzed, 13 % (42 somatically derived + 25 germline derived)/517) were differentially expressed (Table 1) including 31 that were up regulated and 36 that were down-regulated relative to controls (Additional file 7: Table S6).

### Chromosomal translocations are most frequently associated with changes in gene expression

Chromosomal translocations are the physical basis of nearly all gene fusions and we found them to be the most abundant class of variants associated with significant changes in gene expression both for somatically derived (57/115 or 50 %) and germline derived SVs (26/53 or 49 %). Although only 6 % ((573 + 232)/ (4516 + 5518); Fig. 1) of all SVs are associated with translocations, our transcriptional analysis indicates that they are the most likely class of SVs to be associated with changes in gene expression (Table 2; Additional file 2: Figure S5; Additional file 9: Table S8).

**Table 2:** Summary of the number of various functional classes of SVs across multiple structural classes of SVs

	Somatically derived		Germline derived	
svClass	SVs at DNA level	Detected by RNA-Seq / differentially expressed (microarray)	SVs at DNA level	Detected by RNA-Seq / differentially expressed (microarray)
Deletion	92	17	154	16
Insertion	13	2	0	0
Inversion	45	10	17	4
Inv-dupli	86	8	74	5
Tandem-dupli	49	21	7	2
Translocation	398	57	125	26
Transposition	4	0	7	0
**Total**	**677**	**115**	**384**	**53**

## Discussion

An integrated high-throughput computational workflow was employed to accurately detect a remarkably large number (10,034) of SVs in cancerous tissue samples isolated from 6 ovarian cancer patients. This number is larger than previous estimates of the number of SVs in other types of cancer tissues and cell lines [8] possibly due to the greater accuracy afforded by our *de novo* assembly approach and/or because of the exceptional chromosomal instability known to be associated with ovarian cancers [45]. The majority (5518) of these SVs were determined to be of germline origin, reflective of the abundance of naturally occurring SVs believed to be segregating in human populations [46]. An additional large number of the SVs (4516) identified in the ovarian cancer samples were determined to be of somatic origin arising *de novo* in the cancer cell lineage. Somatically derived SVs have recently been reported to constitute a major fraction of somatic tissue genetic variation in humans [47] and our results are consistent with these findings. Since our study was based on the analysis of only six ovarian cancer patients and we employed a stringent set of filtering criteria, our results likely constitute a relatively conservative estimate of SVs that may be associated with OC. Indeed, two recently identified OC gene fusion variants [48, 49] were not among those identified in our study.

While a major fraction of the SVs identified in our study were shown to map to unannotated regions of the genome (gene deserts), the functional significance of these variants is currently unknown. In contrast, intergenic SVs, while constituting only 11 % of the SVs identified in our study, are the basis of gene fusions- a well defined class of SVs previously demonstrated to be of functional significance in the onset and progression of a variety of cancers [6–8].

To our knowledge, ours is the first study to systematically analyze both the presence and expression of SVs in the same panel of cancer patient samples. Among the most notable findings coming out of this comparative analysis is the remarkably low proportion of cancer SVs that are being transcribed. Only 20 % of the gene fusions detected in our DNA-Seq analysis were detectable in the RNA-Seq analysis of the same samples. Remarkably, none of the germline derived gene fusions but all of the somatically derived gene fusions were detectable on the RNA level. This observation suggests the existence of a regulatory mechanism or mechanisms that may effectively suppress older, more established germline SVs segregating in natural populations. Such repression mechanisms may be lost or otherwise rendered less effective in suppressing *de novo* variants arising in cancer cell lineages. Consistent with this possibility is the recent finding that a microRNA (miR-203) that targets and suppresses expression of the BCR-ABL fusion protein is hypermethylated in several hematopoietic tumors including chronic myelogenous leukemias and some lymphoblastic leukemias. Re-expression of this microRNA has been shown to significantly reduce BCR-ABL fusion protein levels and to coincidently inhibit tumor cell proliferation [50]. The relevance of this and/or other regulatory mechanisms to the fact that several gene fusions previously identified as biomarkers of cancer have recently been found to be present in normal healthy individuals [51] remains to be determined.

Further evidence of the importance of the regulation of gene fusions and other SVs in cancer comes from our microarray analyses. We found that only 10–30 % of both germline and somatically derived fusions display a significant change in the expression of those genes involved in the fusion relative to normal controls. In several cases, changes in the expression of the protein coding domains involved in the fusions have been previously associated with cancer onset or progression.

## Conclusions

Collectively, our findings are consistent with the view that gene fusions and other SVs may be significant factors in the onset and progression of ovarian cancer. Our results further suggest that it is not only the occurrence of these variants in cancers but their regulation that contributes to their biological and clinical significance.

## Consent

Written informed consent was obtained from the patients for publication of this report and any accompanying images.

## Additional files

Additional file 1: Table S1. Summary data on processed whole genome sequencing data and detected structural variants from 12 samples [6 control (whole blood), 6 cancer tissue samples]. Additional file 2: Supplemental figures. **Figure S1.** Integrative data analysis workflow for structural variants. The upper workflow summarizes detection and validation of SVs using whole genome sequence data. The bottom left workflow summarizes the detection of fusion-transcripts using RNA-Seq data. The estimation of differential gene-expression using microarray data is summarized in the bottom right of the figure. **Figure S2.** Validation and breakpoint detection. Assembly of a SV call representing a translocation event between two chromosomes (green and orange) is illustrated. SV calls are generated by SVDetect using paired-end signatures (blue and purple boxes represent paired-mates). Reads mapping to the genomic regions surrounding them (black boxes) are assembled using *de novo* assembly that also included initially unmapped reads. Assembled contigs shown here as a black line represent the DNA sequence underlying the translocation event. Contigs are mapped back to the reference genome and if split-mapping is observed, a SV call is considered validated and breakpoints (shown in red) are considered detected. **Figure S3.** The number (percentage) of germline and cancer derived SVs in each of the six patient samples. Circles represent the total number of SVs detected in somatic control (whole blood; blue circle) and cancer (red circle) tissue samples collected from each of 6 ovarian cancer patients. SVs detected in both the somatic and cancer samples were characterized as germline derived while SVs found only in the cancer samples were designated as somatically derived. **Figure S4.** Hierarchical classification of ovarian cancer SVs. Genomic coordinates of SVs and their breakpoint were compared with the reference transcripts that served as the basis of hierarchical classification into functionally relevant classes. The total number of SVs in each class is shown in black font while the numbers in parenthesis represent the distribution of somatically derived (red) and germline derived (green) SVs. **Figure S5.** Genomic distribution of intergenic SVs. **A.** displays the distribution of somatically derived intergenic SVs, **B.** displays the distribution of those somatically derived intergenic SVs that were either transcribed (detected using RNA-Seq) or resulted in differential gene-expression (measured using gene-expression microarrays). Similar distributions for germline derived SVs are shown in **C** and **D**. Each line (blue: translocation, red: deletion, green: tandem-duplication, yellow: inverted-duplication, orange: inversion, grey: insertion, dark grey: transposition) connecting two different chromosomes (outer circle) represents inter-chromosomal and straight lines restricted to a single chromosome represent intra-chromosomal rearrangements. Additional file 3: Table S2. Read-groups in the ovarian WGS data and various cutoffs used for the SV detection. Additional file 4: Table S3. Detailed alignment and annotation information on 14,719 validated SVs from 12 ovarian samples [6 control (whole blood) and 6 ovarian cancer patient samples] analyzed in this study. Additional file 5: Table S4. Table summarizing the potential functional impacts of each class of intergenic SV. Additional file 6: Table S5. Distribution of somatically and germline derived SVs that were characterized as intragenic SVs. SVs were classified based on the location of breakpoints within the same gene. Since intragenic SVs do not create gene fusions, they were not pursued in the RNA-Seq and gene-expression microarray analysis. Additional file 7: Table S6. Somatically and germline derived intergenic SVs that were detected by RNA-Seq or differential gene-expression measured by microarray. Additional file 8: Table S7. Summary of the ovarian samples used to perform the microarray (Affymetrix HT_HG-U133A) gene-expression by TCGA. Additional file 9: Table S8. Detailed distribution of functional classes of intergenic SVs among various structural classes of SVs. Structural classes of SVs are listed vertically in the left-most column, functional classes across the top of the columns.
