# Supplementary material for: Integrated sequence and expression analysis of ovarian cancer structural variants underscores the importance of gene fusion regulation
Source: BMC Med Genomics. 2015 Jul 17;8:40. doi: 10.1186/s12920-015-0118-9 (PMC4504069; doi:10.1186/s12920-015-0118-9)
Supplement: Additional file 2: — Supplemental figures. Figure S1. Integrative data analysis workflow for structural variants. The upper workflow summarizes detection and validation of SVs using whole genome sequence data. The bottom left workflow summarizes the detection of fusion-transcripts using RNA-Seq data. The estimation of differential gene-expression using microarray data is summarized in the bottom right of the figure. Figure S2. Validation and breakpoint detection. Assembly of a SV call representing a translocation event between two chromosomes (green and orange) is illustrated. SV calls are generated by SVDetect using paired-end signatures (blue and purple boxes represent paired-mates). Reads mapping to the genomic regions surrounding them (black boxes) are assembled using de novo assembly that also included initially unmapped reads. Assembled contigs shown here as a black line represent the DNA sequence underlying the translocation event. Contigs are mapped back to the reference genome and if split-mapping is observed, a SV call is considered validated and breakpoints (shown in red) are considered detected. Figure S3. The number (percentage) of germline and cancer derived SVs in each of the six patient samples. Circles represent the total number of SVs detected in somatic control (whole blood; blue circle) and cancer (red circle) tissue samples collected from each of 6 ovarian cancer patients. SVs detected in both the somatic and cancer samples were characterized as germline derived while SVs found only in the cancer samples were designated as somatically derived. Figure S4. Hierarchical classification of ovarian cancer SVs. Genomic coordinates of SVs and their breakpoint were compared with the reference transcripts that served as the basis of hierarchical classification into functionally relevant classes. The total number of SVs in each class is shown in black font while the numbers in parenthesis represent the distribution of somatically derived (red) and germline derived (green) SVs. [file 12920_2015_118_MOESM2_ESM.pdf]

Additional File 2

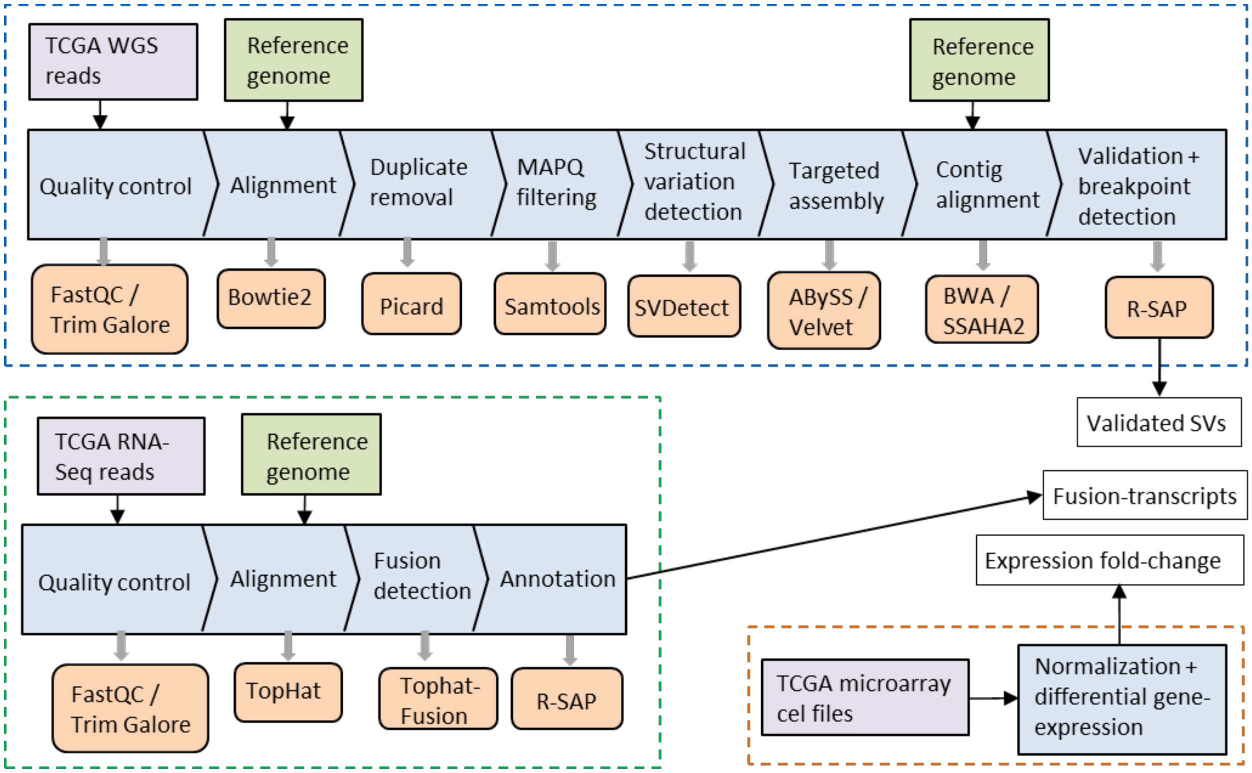

Figure S1 Integrative data analysis workflow for structural variants. The upper workflow summarizes detection and validation of SVs using whole genome sequence data. The bottom left workflow summarizes the detection of fusion-transcripts using RNA-Seq data. The estimation of differential gene-expression using microarray data is summarized in the bottom right of the figure.

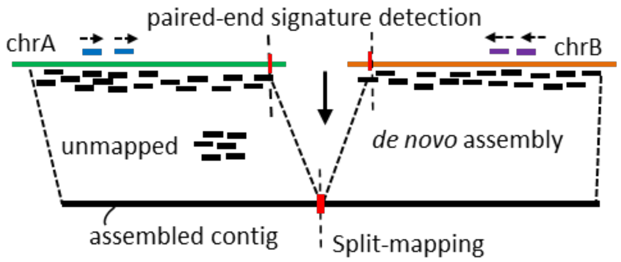

Figure S2 Validation and breakpoint detection. Assembly of a SV call representing a translocation event between two chromosomes (green and orange) is illustrated. SV calls are generated by SVDetect using paired-end signatures (blue and purple boxes represent paired-mates). Reads mapping to the genomic regions surrounding them (black boxes) are assembled using *de novo* assembly that also included initially unmapped reads. Assembled contigs shown here as a black line represent the DNA sequence underlying the translocation event. Contigs are mapped back to the reference genome and if split-mapping is observed, a SV call is considered validated and breakpoints (shown in red) are considered detected.

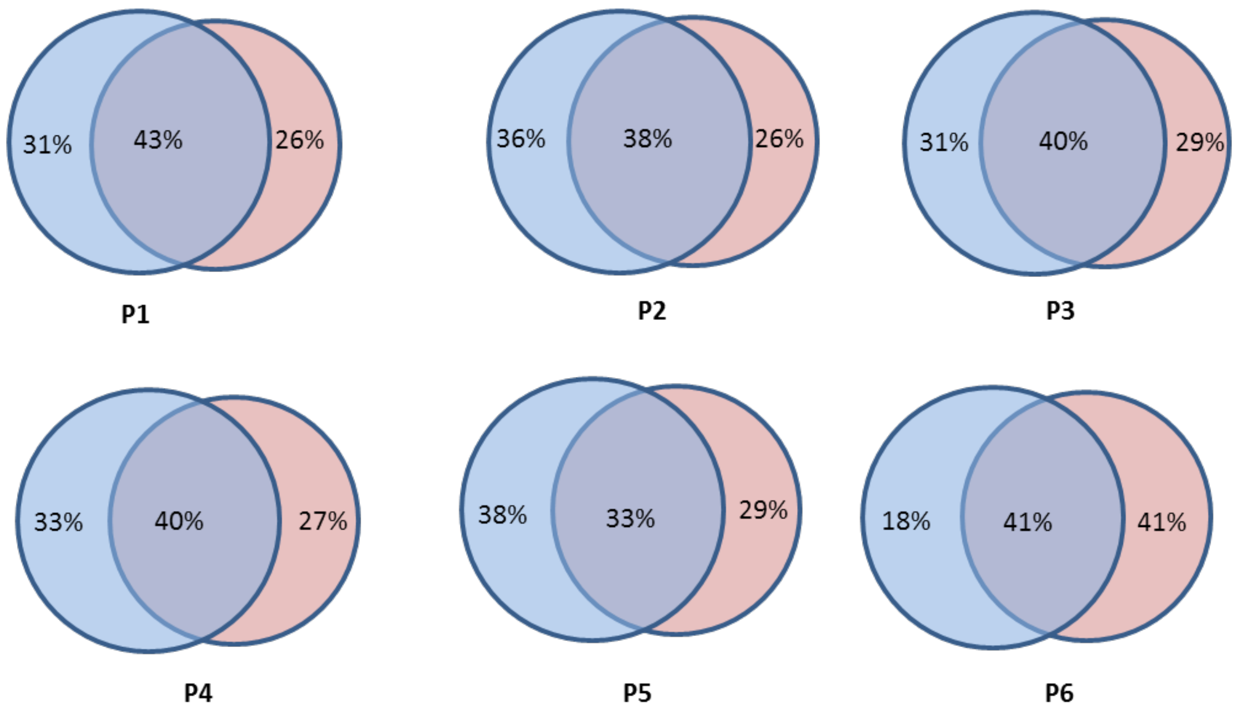

Patient Id	Somatic control	Somatically derived	Germline derived	Total
P1	711	616	986	2313
P2	1793	1316	1876	4985
P3	1414	1331	1859	4604
P4	1555	1238	1844	4637
P5	2126	1635	1866	5627
P6	654	1516	1478	3648

Figure S3 The number (percentage) of germline and cancer derived SVs in each of the six patient samples. Circles represent the total number of SVs detected in somatic control (whole blood; blue circle) and cancer (red circle) tissue samples collected from each of 6 ovarian cancer patients. SVs detected in both the somatic and cancer samples were characterized as germline derived while SVs found only in the cancer samples were designated as somatically derived.

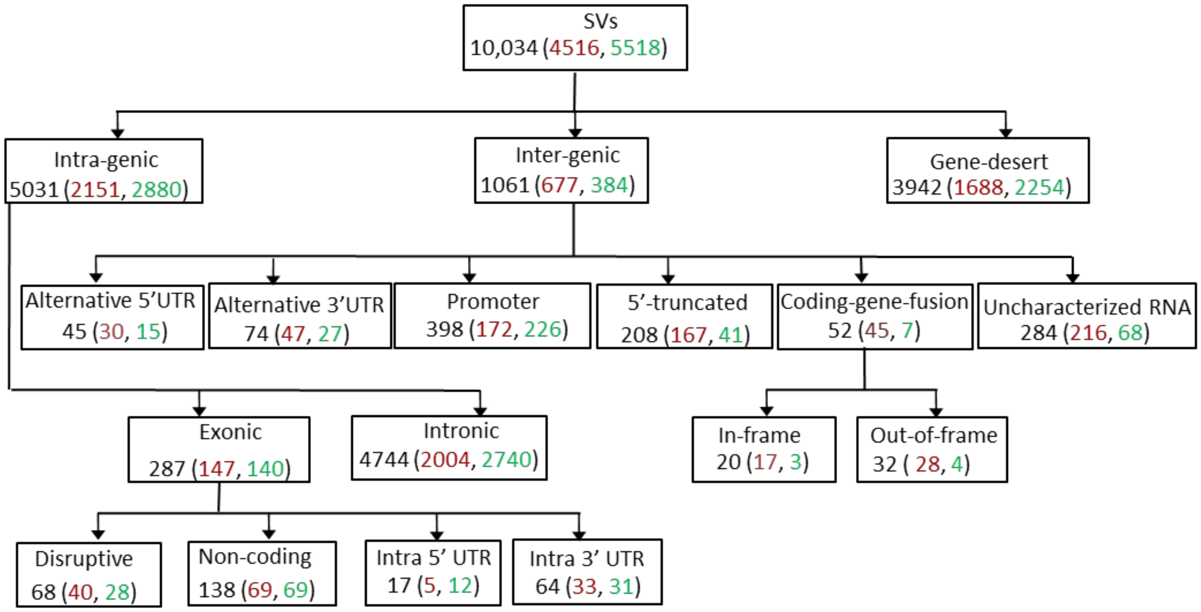

Figure S4 Hierarchical classification of ovarian cancer SVs. Genomic coordinates of SVs and their breakpoint were compared with the reference transcripts that served as the basis of hierarchical classification into functionally relevant classes. The total number of SVs in each class is shown in black font while the numbers in parenthesis represent the distribution of somatically derived (red) and germline derived (green) SVs.

A.

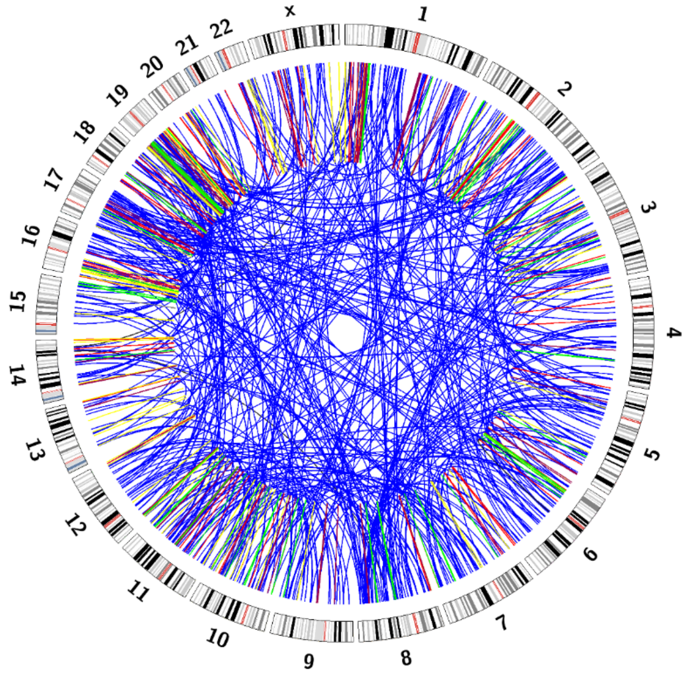
x
1
2
3
4
5
6
7
8
9
10
11
12
13
14
15
16
17
18
19
20
21
22

B.

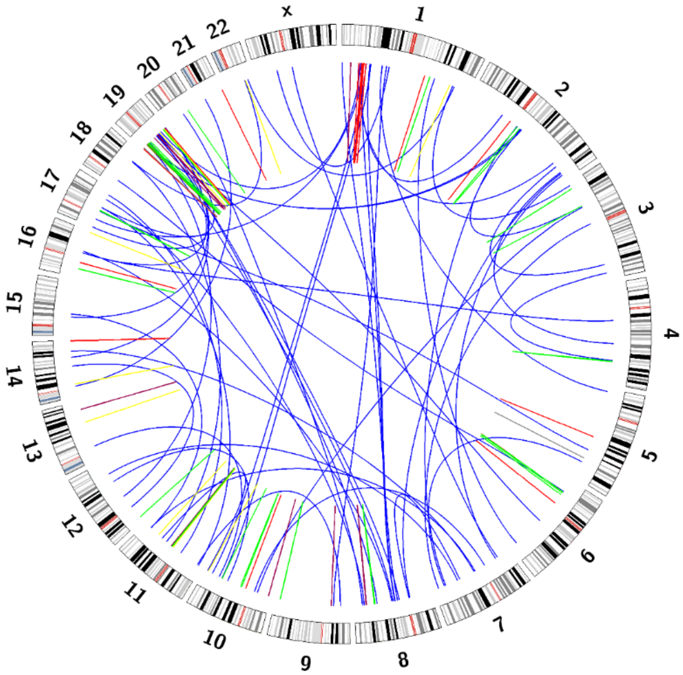
x
1
2
3
4
5
6
7
8
9
10
11
12
13
14
15
16
17
18
19
20
21
22

C.

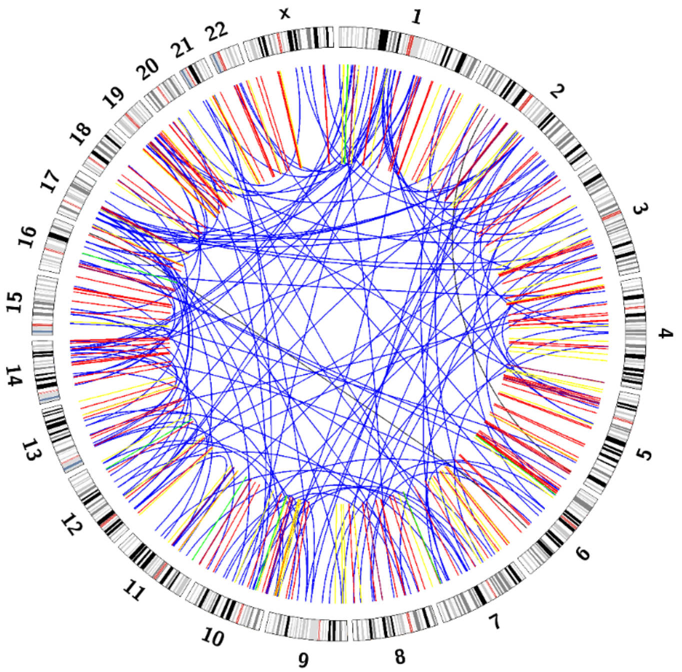

D
.

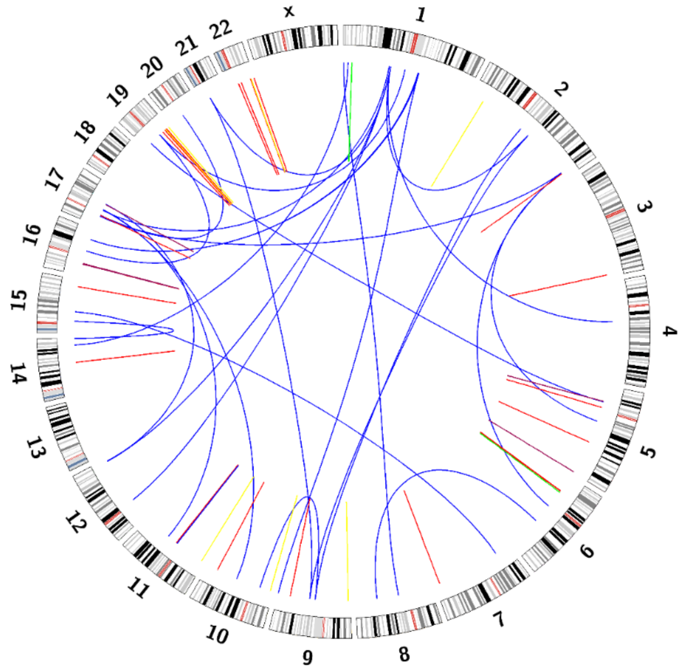

Figure S5 Genomic distribution of inter-genic SVs. A. displays the distribution of somatically derived inter-genic SVs, **B.** displays the distribution of those somatically derived inter-genic SVs that were either transcribed (detected using RNA-Seq) or resulted in differential gene-expression (measured using gene-expression microarrays). Similar distributions for germline derived SVs are shown in **C** and **D**. Each line (blue: translocation, red: deletion, green: tandem-duplication, yellow: inverted-duplication, orange: inversion, grey: insertion, dark grey: transposition)

connecting two different chromosomes (outer circle) represents inter-chromosomal and straight lines restricted to a single chromosome represent intra-chromosomal rearrangements.
