# Supplementary material for: Integrated sequence and expression analysis of ovarian cancer structural variants underscores the importance of gene fusion regulation
Source: BMC Med Genomics. 2015 Jul 17;8:40. doi: 10.1186/s12920-015-0118-9 (PMC4504069; doi:10.1186/s12920-015-0118-9)
Supplement: Additional file 5: Table S4. — Table summarizing the potential functional impacts of each class of intergenic SV. [file 12920_2015_118_MOESM5_ESM.doc]

**Supplemental Table S4 Table summarizing the potential functional impacts of each class of inter-genic SV.**
