# Supplementary material for: Integrated sequence and expression analysis of ovarian cancer structural variants underscores the importance of gene fusion regulation
Source: BMC Med Genomics. 2015 Jul 17;8:40. doi: 10.1186/s12920-015-0118-9 (PMC4504069; doi:10.1186/s12920-015-0118-9)
Supplement: Additional file 6: Table S5. — Distribution of somatically and germline derived SVs that were characterized as intragenic SVs. SVs were classified based on the location of breakpoints within the same gene. Since intragenic SVs do not create gene fusions, they were not pursued in the RNA-Seq and gene-expression microarray analysis. [file 12920_2015_118_MOESM6_ESM.doc]

**Supplemental Table S5 Distribution of somatically and germline derived SVs that were characterized as intra-genic SVs.** SVs were classified based on the location of breakpoints within the same gene. Since intra-genic SVs do not create gene-fusions, they were not pursued in the RNA-Seq and gene-expression microarray analysis.
