# Supplementary material for: Integrated sequence and expression analysis of ovarian cancer structural variants underscores the importance of gene fusion regulation
Source: BMC Med Genomics. 2015 Jul 17;8:40. doi: 10.1186/s12920-015-0118-9 (PMC4504069; doi:10.1186/s12920-015-0118-9)
Supplement: Additional file 8: Table S7. — Summary of the ovarian samples used to perform the microarray (Affymetrix HT_HG-U133A) gene-expression by TCGA. [file 12920_2015_118_MOESM8_ESM.doc]

**Supplemental Table S7 Summary of the ovarian samples used to perform the microarray (Affymetrix HT_HG-U133A) gene-expression by TCGA.**
